# Antimicrobial Activity and Urease Inhibition of Schiff Bases Derived from Isoniazid and Fluorinated Benzaldehydes and of Their Copper(II) Complexes

**DOI:** 10.3390/molecules21121742

**Published:** 2016-12-17

**Authors:** Ladislav Habala, Samuel Varényi, Andrea Bilková, Peter Herich, Jindra Valentová, Jozef Kožíšek, Ferdinand Devínsky

**Affiliations:** 1 Department of Chemical Theory of Drugs, Faculty of Pharmacy, Comenius University, Kalinčiakova 8, SK-832 32 Bratislava, Slovakia; varenyi@fpharm.uniba.sk (S.V.); herich@fpharm.uniba.sk (P.H.); valentova@fpharm.uniba.sk (J.V.); devinsky@fpharm.uniba.sk (F.D.); 2 Department of Cellular and Molecular Biology of Drugs, Faculty of Pharmacy, Comenius University, Kalinčiakova 8, SK-832 32 Bratislava, Slovakia; bilkova@fpharm.uniba.sk; 3 Department of Physical Chemistry, Faculty of Chemical and Food Technology, Slovak University of Technology, Radlinského 9, SK-812 37 Bratislava, Slovakia; jozef.kozisek@stuba.sk

**Keywords:** copper(II) complexes, urease inhibitors, antimicrobial activity, fluorine, isoniazid

## Abstract

In order to evaluate the influence of substitution on biological properties of Schiff bases and their metal complexes, a series of differently substituted fluorine-containing Schiff bases starting from the drug isoniazid (isonicotinylhydrazide) were prepared and their structures were established by single-crystal X-ray diffraction. Also, four copper(II) complexes of these Schiff bases were synthesized. The prepared compounds were evaluated for their antimicrobial activity and urease inhibition. Two of the Schiff bases exerted activity against *C. albicans*. All copper(II) complexes showed excellent inhibitory properties against jack bean urease, considerably better than that of the standard inhibitor acetohydroxamic acid.

## 1. Introduction

Over the last few decades, the research field of medicinal inorganic chemistry has experienced continuous growth. The application areas encompass antibacterial, anti-inflammatory, and anticancer therapies [1], among others. The early metallodrugs often exerted their biological activity via direct covalent binding to the target biomolecule (e.g., cisplatin). Recently, the research increasingly concentrates on metallodrug candidates acting by enzyme inhibition, in other words, by reversible binding to the active centre of an enzyme, thus influencing its biological function [2,3,4].

One of the potential targets for new metallodrugs is urease, an enzyme catalysing hydrolysis of urea to ammonia and carbon dioxide [5]. Activity of this enzyme has been implicated in the pathogenesis of several diseases [6,7,8]. The most prominent example is the urease found in *Helicobacter pylori*, a bacterium presumably involved in several pathological conditions (gastric ulcers, gastritis, gastric cancer) [9,10,11]. This bacterium is able to colonize the acidic milieu of the stomach by increasing its pH in the course of ammonia production. Another important pathogen expressing urease is *Mycobacterium tuberculosis* [12]. Thus, the inhibition of urease represents an effective way to combat these pathogens.

A number of urease inhibitors have been reported [13]. Besides solely organic substances, a very efficient group of urease inhibitors is formed by metal complexes. The “free” metal ions themselves (i.e., their aqua complexes) show marked urease inhibition [14]. The aquated transition metal ions were found to inhibit urease in the order Cu^2+^ > Ni^2+^ > Co^2+^ ≅ Zn^2+^ [15]. The complexes of these metals follow roughly the same order of efficiencies, although the inhibitory activity depends to a large degree on the nature of the ligands and the overall structure of the complexes. Copper(II) complexes are among the most efficient urease inhibitors [16]. Besides this, they exhibit a number of other interesting biological activities, for example, anticancer activity [17].

As part of our research, we are interested in the inhibitory properties of metal complexes towards enzymes, such as urease [16] or protein tyrosine phosphatase [18,19]. Fluorine-containing compounds constitute a class of compounds with unique pharmacological properties [20]. Schiff bases are another interesting type of substance, both as organic substances and as ligands for the synthesis of complexes [21,22]. There have been several reports [23,24,25,26,27] on transition metal complexes of Schiff bases derived from isoniazid, a highly efficient tuberculostatic [28]. The aim of our study was to investigate hydrazine derivates of isoniazid with fluoro and trifluoromethyl substituted aromatic aldehydes, as well as their copper(II) complexes. The ligands were synthesized according to established synthetic procedures. The prepared substances were screened for their urease inhibition against jack bean urease and also for their antimicrobial properties, with the intent to evaluate the effect of the substituents and their location on the bioactivity of the prepared substances. Also, we successfully recorded X-ray structural data for the Schiff bases as an important tool for evaluating various pharmacological parameters. This publication utilizes research results of the CEBV project (Centrum Excelencie Bezpečnostného Výskumu/Centre of Excellence of Security Research), ITMS: 26240120034.

## 2. Results and Discussion

### 2.1. Synthesis

**Scheme 1:**
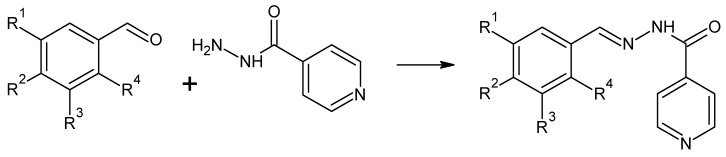
Synthesis of the Schiff bases **L1**–**L6** (**L1**: R^2^ = CF_3_, R^1^ = R^3^ = R^4^ = H; **L2**: R^4^ = CF_3_, R^1^ = R^2^ = R^3^ = H; **L3**: R^2^ = F, R^1^ = R^3^ = R^4^ = H; **L4**: R^1^ = F, R^4^ = OH, R^2^ = R^3^ = H; **L5**: R^3^ = F, R^4^ = OH, R^1^ = R^2^ = H; **L6**: R^1^ = R^2^ = R^3^ = R^4^ = H).

The Schiff base ligands were prepared by condensation reactions between isoniazid and appropriately substituted benzaldehydes (Scheme 1). The reaction is straightforward and provides the products in good to excellent yields. Use of methanol-chloroform mixture as a reaction medium often leads directly to X-ray quality crystals. Alternatively, the monocrystals can be obtained upon recrystallization from ethanol and they generally crystallize in the form of monohydrates as white needles stable in air and are soluble in polar organic solvents.

The synthesis of the copper(II) complexes was carried out by combining ethanolic solutions of the particular Schiff base ligand with copper(II) acetate in a metal:ligand molar ratio of 2:1. The complexes are green-coloured solids and are poorly soluble in common organic solvents with the exception of dimethyl sulfoxide (DMSO) and dimethylformamide (DMF). The attempts to grow crystals suitable for X-ray structure analysis were unsuccessful. The complexes were non-electrolytes as proven by the measurement of their conductivities in 1 mM DMSO solutions (molar conductivities ranging from 7.2 to 26.9 S·mol^−1^·cm^2^). The composition of the complexes corresponds to [Cu(L*^n^*-H)_2_], as seen from the results of elemental analysis and infrared spectroscopy.

### 2.2. Analytical Characterization of the Compounds

The products were characterized by appropriate analytical methods—elemental analysis, NMR, and infrared spectroscopy. The characteristic infrared (IR) vibrational bands are listed in the Experimental part and are in accordance with the expected values [29]. Infrared spectra of the Schiff bases contain a strong and sharp peak near 1670 cm^−1^, characteristic of the valence vibration of the carbonyl group (amide I). Signals from the valence vibrations of the azomethine group can be seen around 1660 cm^−1^. The medium peak near 1415 cm^−1^ accounts for the deformation vibration of the C–N bond of amide (amide II). The strong and sharp peak near 1200 cm^−1^ originates from the valence vibration of the C–F bond. The very strong peak near 750 cm^−1^ corresponds to the deformation vibration of the aromatic C–H bond. The N–N bond causes infrared absorption in the ligand spectra near 850 cm^−1^. Hydroxyl containing Schiff base ligands also show a very strong peak near 1280 cm^−1^ for phenol C–O valence vibration.

In the infrared spectrum of the ligands, a signal of valence vibration of the N–H bond can be detected near 3200 cm^−1^. The disappearance of this signal in the infrared spectra of the complexes clearly indicates deprotonation at the nitrogen due to enolization of the adjoining carbonyl group in the course of complex formation. The strong signal of the carbonyl group found in the range of 1667–1691 cm^−1^ disappears upon coordination and the new medium-strong signals corresponding to enolic C–O stretching vibration appear at 1056–1066 cm^−1^. Vibrational bands of the O–H group originating from crystal water are found in some ligands. These bands also disappear in the course of the coordination to the central atom. Absence of signals of the water molecules in the infrared spectra disproves the possibility of any additional aqua ligands present in the copper complexes. Thus, the structure of the complexes seems to correspond to the general formula [ML_2_] with a square-planar coordination at the central atom, unlike the complexes reported in [23,25], supposed to possess two extra aqua ligands and an octahedral geometry. This assumption is further supported by the results of elemental analysis of the Cu(II) complexes, confirming the expected composition with metal-to-ligand ratio 1:2 and the absence of aqua ligands in the structure of the complexes. A definite assignment of particular coordination geometry to the complexes would require the preparation of a single crystal and its X-ray structure analysis, which has not yet met with success, mainly due to the problems with solubility.

### 2.3. X-ray Structures of the Schiff Bases

**Figure 1:**
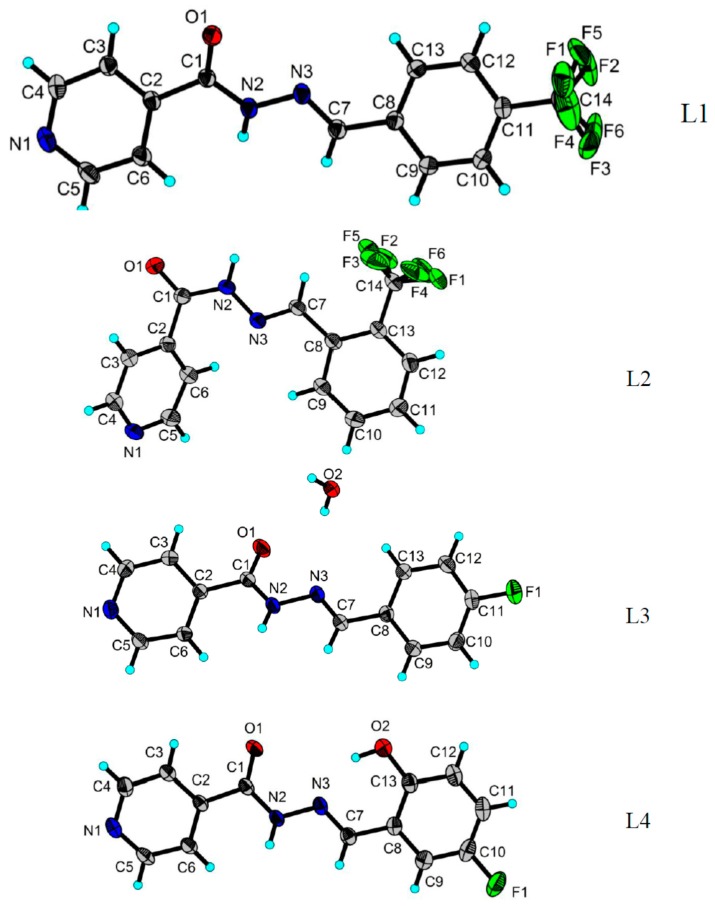
The molecular structure of ligands (**L1**–**L4**), showing the atom-labelling scheme. Displacement ellipsoids are drawn at the 50% probability level.

The three ligands **L1**, **L2**, and **L4** crystallized in the monoclinic P2_1_/n (for **L1**) and P2_1_/c (for **L2** and **L4**) space groups (No. 14). The ligand **L3** crystallized in the orthorhombic P2_1_2_1_2_1_ space group (No. 19). All four ligands crystallized as one independent molecule in the asymmetric unit. The molecular structures for (**L1**–**L4**) are shown in Figure 1.

**Figure 2:**
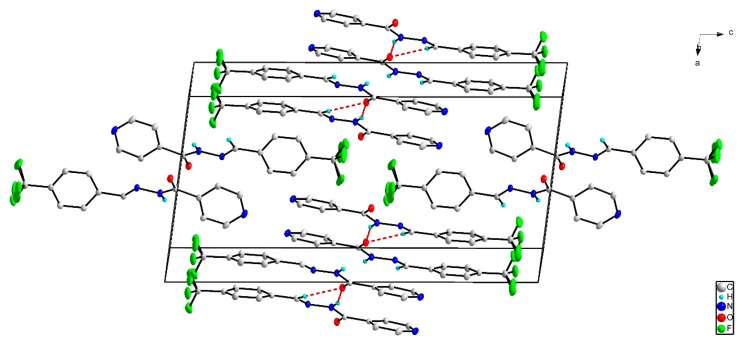
The crystal packing of ligand **L1**, viewed along the b axis. Red dashed lines indicate hydrogen interactions.

**Figure 3:**
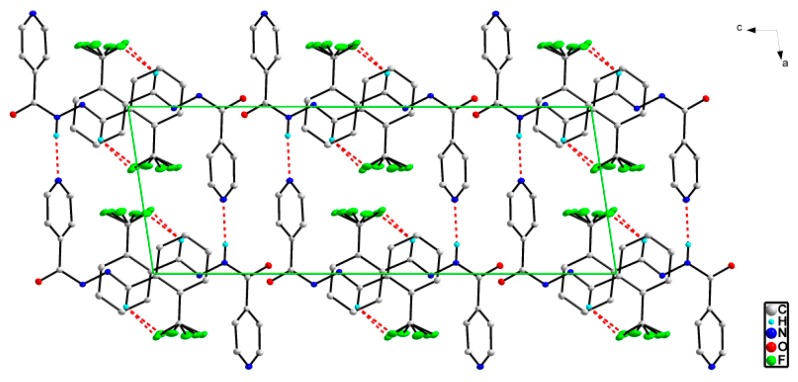
The crystal packing of ligand **L2**, viewed along the b axis. Red dashed lines indicate hydrogen interaction.

**Figure 4:**
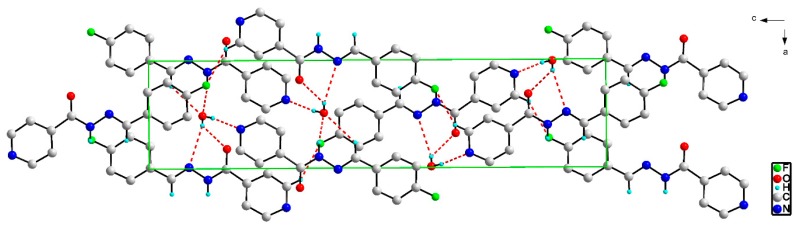
The crystal packing of ligand **L3**, viewed along the b axis. Red dashed lines indicate hydrogen interactions.

**Figure 5:**
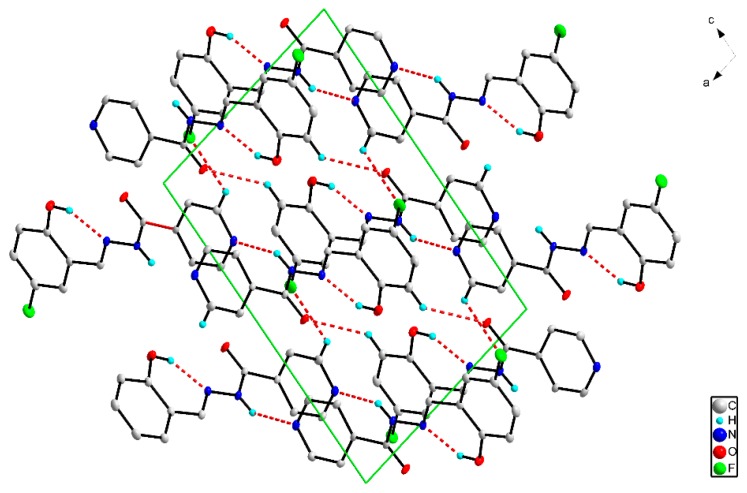
The crystal packing of ligand **L4**, viewed along the b axis. Red dashed lines indicate hydrogen interactions.

The molecule for all ligands comprises three chemical moieties: pyridine, benzene (substituted with –OH, –F and –CF_3_), and hydrazone. The ligand molecules are not planar, with the angles between the benzene and pyridine rings being 4.05° for **L1**, 51.62° for **L2**, 11.55° for **L3**, and 10.19° for **L4**. C–N and N–N bond distances (Table 1) are shorter than a typical C–N, N–N single bond 1.405(6) Å [30,31,32], indicating significant π delocalization along the NNC(O)C moiety. The shorter C–N and N–N bond distances (Table 1) were observed in other compounds; for C–N, the bond distance is in the range of 1.343(2) Å–1.362(6) Å, and for N–N, the bond distance is in the range of 1.359(5) Å–1.384(3) Å [33,34,35,36,37,38,39,40,41,42]. The N=C bond distances (Table 1), are typical for a double bond. For all ligands, the central torsion angles C1–N2–N3–C7 are listed in Table 1. The crystal structures of ligands **L1**–**L4** are stabilized by a system of intermolecular and intramolecular (for **L2** and **L4**) hydrogen interactions (Table 2, Figure 2, Figure 3, Figure 4 and Figure 5).

### 2.4. Bioactivity of the Ligands and Complexes

Antimicrobial activity of the Schiff bases and the Cu(II) complexes was evaluated in vitro against Gram-negative (*E. coli*) and Gram-positive (*S. aureus*) bacterial strains, and against *C. albicans*. Also, the standard drugs ciprofloxacin and isoniazid were co-assayed for comparison. The results are summarized in Table 3 as minimum inhibitory concentration (MIC) values. Two Schiff bases (**L1** and **L4**) showed marked activity against *C. albicans* (MIC 0.037 mM and 0.048 mM, respectively). The Schiff base **L4** also exhibited weak activity against *E. coli* (MIC 1.55 mM). Among the copper(II) complexes, only **Cu**-**L6** showed any noteworthy activity (against *S. aureus*, MIC 0.76 mM). The hydrophobic fluoro and trifluoromethyl groups confer marked lipophilic properties to the molecules of ligands. Upon complexation, the lipophilicity becomes even more pronounced, as the polar hydrophilic groups are used in coordination to the central atom and hidden inside the complex molecule, while the hydrophobic moieties are exposed to the environment. Although this could be expected to increase the penetration through biomembranes, a marked increase in activity upon complexation was not observed. It is possible that high lipophilicity might lead to decreased mobility of the substances in aqueous cell media and impair the interaction with potential targets in the cell. In the two ligands containing hydroxyl (**L4** and **L5**), the Schiff base with fluoro substituent in para position to the hydroxyl group had significantly higher activity than with the same group in the ortho position. Also, in trifluoromethyl containing ligands (**L1** and **L4**), para substitution shows a positive effect on antimicrobial activity compared to the ortho substitution.

Jack bean urease is a urease isolated from *Canavalia ensiformis* (Jack bean). Because of their highly conserved structure, ureases from different sources share similar structural features, and conclusions may be drawn concerning the properties of the other ureases (such as the *Helicobacter pylori* urease). The inhibition of Jack bean urease by Schiff bases, copper(II) complexes and the standard substance acetohydroxamic acid was studied, and the results are expressed in the form of their IC_50_ values (Table 4). All Cu(II) complexes showed excellent inhibitory properties, whereas the ligands exerted only weak activity. The best activity (0.49 ± 0.01 μM) was observed in the Cu(II) complex of *N*-[[(4-(trifluoromethyl)phenyl]methylidene]pyridine-4-carbohydrazide (**Cu**-**L1**). The inhibition is clearly influenced by the structure of the ligand, particularly on the substitution of the phenyl ring, as the overall structure of the complex is analogous and the low electric conductivity of the DMSO solutions of the complexes renders improbable the dissociation of the complexes and the formation of charged Cu^2+^ species to any significant degree. Nevertheless, at this stage, it is difficult to draw clear conclusions regarding the structure–activity relationships for the inhibition of urease by these copper(II) complexes. More extensive studies on the mechanism of urease inhibition by metal complexes are needed to accomplish this, including data on lipophilicity and stability constants of the complexes, amongst others.

## 3. Materials and Methods

### 3.1. General

All chemicals purchased from commercial sources were of analytical grade and were used without further purification. The chemicals 3-fluoro-2-hydroxybenzaldehyde and 5-fluoro-2-hydroxybenzaldehyde were prepared by a modified procedure according to Ferguson et al. [43]. Cell cultures were purchased from the Czech National Collection of Type Cultures. Infrared spectra were recorded using the ATR (attenuated total reflectance) technique on a Nicolet 6700 FT-IR spectrometer from Thermo Scientific (Waltham, MA, USA) in the 600–4000 cm^−1^ range. Elemental analysis was performed with the help of a Flash2000 instrument from Thermo Scientific. ^1^H-, ^13^C- and ^19^F-NMR spectra were measured on a Varian Mercury 300 MHz instrument (Palo Alto, CA, USA) using DMSO-*d*_6_ as solvent and TMS (tetramethylsilane) as internal standard. Melting points were determined on a Büchi B-540 apparatus (Büchi Labortechnik AG, Flawil, Switzerland). The conductivities of the copper(II) complexes were evaluated in DMSO solutions (10^−3^ mol/L, 25 °C) on a WTW LF 530 conductometer (Weilheim, Germany).

### 3.2. Synthesis of the Schiff Bases

General procedure for the synthesis of the Schiff bases. The ligand, *N*-[[(4-(trifluoromethyl)phenyl]methylidene]pyridine-4-carbohydrazide (**L1**), was prepared in the following way. A 1.37 g portion (10.0 mmol) of isoniazid and 1.74 g (10.0 mmol) of 4-trifluoromethylbenzaldehyde were dissolved in 40 mL of methanol-chloroform mixture (1:1 *v*/*v*). The solution was then refluxed for 80 min and then left to freely crystallise at room temperature. After three days, colourless crystals suitable for X-ray structure analysis were obtained. They were filtered off, washed with ether, and sucked dry in open air. If the crystals were of unsatisfactory quality for crystallography, they were recrystallized from methanol-chloroform or methanol-dichloromethane mixtures. The other ligands were prepared in an analogous way.

*N-[[(4-(trifluoromethyl)phenyl]methylidene]pyridine-4-carbohydrazide* (**L1**). Yield 91% (white needle-like crystals); m.p. 198–200 °C. IR (neat, cm^−1^): 1667 (vs, C=O, amide I), 1172 (vs, C–F), 1119 (vs, C–F), 766 (s, C–H, arom.), 689 (s, C–H, arom.). ^1^H-NMR (DMSO-*d*_6_, δ, ppm): 7.83–7.86 (m; 4H; aromatic); 8.00 (d; 2H; *J* = 8.1 Hz; aromatic); 8.54 (s; 1H; azomethine); 8.82 (d; 2H; *J* = 6 Hz; aromatic); 12.27 (s; 1H; N–H). ^13^C-NMR (DMSO-*d*_6_, δ, ppm): 121.54 (CF_3_); 125.74; 127.84; 137.97; 140.23 (C=N); 147.21; 150.36; 161.86 (C=O). ^19^F-NMR (282.3 MHz, DMSO-*d*_6_, δ, ppm): −61.20 (s). Elemental anal. calcd. for C_14_H_10_N_3_F_3_O (293.25): C 57.34, H 3.44, N 14.33; found: C 57.01, H 3.57, N 14.40.

*N-[[(2-trifluoromethyl)phenyl]methylidene]pyridine-4-carbohydrazide* (**L2**). Yield 61% (white needle-like crystals); m.p. 210–212 °C. IR (neat, cm^−1^): 3078 (w, C–H, arom.), 1672 (vs, C=O, amide I), 1410 (w, C–N, amide II), 1115 (vs, C–F), 770 (vs, C–H, arom.). ^1^H-NMR (DMSO-*d*_6_, δ, ppm): 7.68 (t; 1H; *J* = 7.2 Hz; *J* = 7.5 Hz; aromatic); 7.68–7.78 (m; 4H; aromatic); 8.27 (d; 1H; *J* = 7.8 Hz; aromatic); 8.82 (d; 2H; *J* = 6.0 Hz; aromatic); 8.86 (d; 1H; *J* = 2.1 Hz; azomethine); 12.38 (s; 1H; N–H). ^13^C-NMR (DMSO-*d*_6_, δ, ppm): 122.02 (CF_3_); 126.45; 127.47; 130.91; 132.34; 133.43; 140.59 (C=N); 144.42; 150.86; 162.34 (C=O). ^19^F-NMR (282.3 MHz, DMSO-*d*_6_, δ, ppm): −56.71 (s). Elemental anal. calcd. for C_14_H_10_N_3_F_3_O (293.25): C 57.34 H 3.44 N 14.33; found: C 57.38 H 3.41 N 14.62.

*N-[(4-fluorophenyl)methylidene]pyridine-4-carbohydrazide monohydrate* (**L3**). Yield 75% (white needle-like crystals); m.p. 192–193 °C. IR (neat, cm^−1^): 1685 (m, C=O, amide I), 1655 (vs, C=N, azomethine), 1232 (s, C–F), 836 (s, C–H, arom.), 689 (vs, C–H, arom.). ^1^H-NMR (DMSO-*d*_6_, δ, ppm): 7.32 (td; 3H; *J* = 2.1 Hz; *J* = 6.9 Hz; aromatic); 7.85–7.80 (m; 3H; aromatic); 8.47 (s; 1H; azomethine); 8.80 (dd; 2H; *J* = 1.8 Hz; *J* = 2.7 Hz; *J* = 4.5 Hz; aromatic); 12.08 (s; 1H; O–H). ^13^C-NMR (DMSO-*d*_6_, δ, ppm): 116.32; 116.61; 121.99; 129.90; 130.02; 131.10; 140.91 (C=N); 148.36; 150.82; 162.09 (C=O); 165.42 (C–F). ^19^F-NMR (282.3 MHz, DMSO-*d*_6_, δ, ppm): −110.04 to −110.54 (m). Elemental anal. calcd. for C_13_H_10_N_3_FO·H_2_O (311.25): C 59.77 H 4.63 N 16.08; found C 59.42 H 5.22 N 16.62.

*N-[(5-fluoro-2-hydroxyphenyl)methylidene]pyridine-4-carbohydrazide* (**L4**). Yield 43% (white needle-like crystals); m.p. 250–252 °C. IR (neat, cm^−1^): 2933 (w, C–H, arom.), 1683 (vs, C=O, amide), 1556 (s, N–H, amide II), 1285 (vs, C–F), 1261 (s, C–O, phenolic), 781 (s, C–H, arom.), 682 (s, C–H, arom.). ^1^H-NMR (DMSO-*d*_6_, δ, ppm): 6.97 (q; 1H; *J* = 4.5 Hz; aromatic); 7.20 (td; 1H; *J* = 3.3 Hz; *J* = 9 Hz; *J* = 11.7 Hz; aromatic); 7.48 (dd; 1H; *J* = 3 Hz; *J* = 3.3 Hz; *J* = 9.3 Hz; *J* = 9.6 Hz; aromatic); 7.85 (dd; 2H; *J* = 1.5 Hz; *J* = 4.5 Hz; aromatic); 8.67 (s; 1H; azomethine); 8.81 (dd; 2H; *J* = 1.5 Hz; *J* = 4.5 Hz; aromatic); 10.86 (s; 1H; O–H); 12.32 (s; 1H; O–H). ^13^C-NMR (DMSO-*d*_6_, δ, ppm): 113.78; 114.11; 118.10; 118.20; 118.71; 119.03; 120.24; 120.34; 121.99; 123.29; 140.43 (C=N); 147.36; 147.39; 150.11; 150.87; 154.11; 154.09; 154.30 (C–O); 157.41 (C–F); 161.95 (C=O). ^19^F-NMR (282.3 MHz, DMSO-*d*_6_, δ, ppm): −124.73 (s); −124.93 to −125.01 (m). Elemental anal. calcd. for C_13_H_10_N_3_O_2_F (259.24): C 60.23 H 3.89 N 16.21; found C 59.96 H 3.73 N 16.46.

*N-[(3-fluoro-2-hydroxyphenyl)methylidene]pyridine-4-carbohydrazide* (**L5**). Yield 38% (white needle-like crystals); m.p. 276–278 °C. IR (neat, cm^−1^): 2995 (w, C–H, arom.), 1680 (vs, C=O, amide I), 1418 (m, C–N, amide II), 1278 (vs, C–O), 1244 (vs, C–F, arom.), 733 (vs, C–H, arom.), 691 (vs, C–H, arom.). ^1^H-NMR (DMSO-*d*_6_, δ, ppm): 6.90–6.97 (m; 1H; aromatic); 7.31 (td; *J* = 1.5 Hz; *J* = 6.6 Hz; *J* = 6.9 Hz; 1H; aromatic); 7.46 (d; *J* = 8.1 Hz; 1H; aromatic); 7.86 (q; *J* = 1.5 Hz; *J* = 3 Hz; 2H; aromatic); 8.7 (s; 1H; azomethine); 8.82 (d; *J* = 5.1 Hz; 2H; aromatic); 11.38 (s; 1H; O–H); 12.42 (s; 1H; O–H). ^13^C-NMR (DMSO-*d*_6_, δ, ppm): 118.14; 118.37; 119.77; 119.86; 121.74; 121.99; 125.12; 125.16; 140.26 (C=N); 145.66; 145.84; 148.73; 148.78; 149.86; 150.90 (C–F); 153.06 (C–O); 161.96 (C=O). ^19^F-NMR (282.3 MHz, DMSO-d_6_, δ, ppm): −135.96 (m); −136.65 (dd; *J* = 4.8 Hz; *J* = 5.65 Hz). Elemental anal. calcd. for C_13_H_10_N_3_O_2_F (259.24): C 60.23 H 3.89 N 16.21; found C 59.33 H 3.84 N 15.93.

*N-(benzylidene)pyridine-4-carbohydrazide* (**L6**). Yield 79% (white needle-like crystals); m.p. 198–201 °C. IR (neat, cm^−1^): 3199 (w, N–H), 3026 (w, C–H arom.), 1691 (vs, C=O, amide), 1565 (s, N–H, amid II), 686 (vs, C–H, arom.). ^1^H-NMR (DMSO-*d*_6_, δ, ppm): 7.49–7.46 (m; 3H; aromatic); 7.84–7.74 (m; 4H; aromatic); 8.48 (s; 1H; azomethine); 8.80 (d, 2H, *J* = 6 Hz, aromatic); 12.06 (s, 1H, N–H). ^13^C-NMR (DMSO-*d*_6_, δ, ppm): 116.44; 118.69; 119.44; 121.46; 121.60; 129.15; 129.26; 131.75; 139.98 (C=N); 148.89; 149.02; 150.33; 150.44; 157.47; 161.33 (C=O). Elemental anal. calcd. for C_13_H_11_N_3_O (225.25): C 69.32 H 4.92 N 18.66; found C 68.75 H 4.78 N 18.54.

### 3.3. Synthesis of the Complexes

General procedure for the synthesis of the copper(II) complexes. The Schiff base ligand *N*-[(4-fluorophenyl)methylidene]pyridine-4-carbohydrazide (**L3**, 0.243 g; 1.0 mmol) was dissolved in 50 mL ethanol and the solution was heated to 50 °C. Then, a solution of copper(II) acetate monohydrate (0.5 mmol; 0.100 g) in 20 mL ethanol was added dropwise under constant stirring. In the course of its addition, a green precipitate started to appear. The reaction mixture was stirred at the same temperature for 2 h. After this, the mixture was allowed to cool to room temperature and the product was isolated by filtration on a glass filter crucible, washed several times with water and ethanol, and dried in vacuum to yield a green solid complex substance (**Cu-L3**). The remaining complexes were prepared in the same way.

*Cu(L3-H)_2_]* (**Cu-L3**) Yield 93% (green powder). IR (neat, cm^−1^): 1520 (s, C=N), 1061 (m, enolic C–O), 823 (s, N–N). Elemental anal. calcd. for C_26_H_18_N_6_CuF_2_O_2_ (259.24): C 56.98 H 3.31 N 15.33; found C 56.45 H 3.36 N 14.97.

*[Cu(L1-H)_2_]* (**Cu-L1**). Yield 78% (green powder). IR (neat, cm^−1^): 1519 (s, C=N), 1066 (m, enolic C–O), 828 (s, N–N). Elemental anal. calcd. for C_28_H_18_N_6_F_6_O_2_Cu (648.02): C 51.90 H 2.80 N 12.97; found C 50.91 H 2.89 N 12.40.

*[Cu(L2-H)_2_]* (**Cu-L2**). Yield 95% (green powder). IR (neat, cm^−1^): 1511 (s, C=N), 1059 (m, enolic C–O), 854 (s, N–N). Elemental anal. calcd. for C_28_H_18_N_6_F_6_O_2_Cu (648.02): C 51.90 H 2.80 N 12.97; found C 51.45 H 2.66 N 12.73.

*[Cu(L6-H)_2_]* (**Cu-L6**). Yield 92% (green powder). IR (neat, cm^−1^): 1513 (s, C=N), 1056 (m, enolic C–O), 843 (s, N–N). Elemental anal. calcd. for C_26_H_20_N_6_O_2_Cu (512.02): C 60.99 H 3.94 N 16.41; found 60.29 H 3.84 N 16.25.

### 3.4. X-ray Single-Crystal Structure Determination

All X-ray measurements were performed on an Oxford Diffraction Gemini R four-circle diffractometer (Oxford Diffraction Ltd.: Abingdon, UK) with CrysAlis [44], using Mo-*Kα* radiation for **L1**, **L3**, **L4**, and Cu-*Kα* radiation for **L2** at 293(1) K. Data reduction was done by *CrysAlis RED* [44]. The structure was solved by the charge-flipping algorithm superflip [45] using *OLEX2* [46]. Refinement was carried out on F^2^ and scattering factors incorporated in *SHELXL-2013* [47] were used. All non-hydrogen atoms were refined with anisotropic thermal parameters. Crystal data for **L1**, **L2**, **L3**, **L4**, data collection procedures, structure determination, and refinement are summarized in Table 5 and Figure 2. The positions of all other H atoms were geometrically optimized and constrained to ride on their parent atoms, with bond length C–H = 0.93 Å (aromatic), N–H = 0.86 Å (hydrazide for **L1**, **L3**, **L4**), N–H = 0.89 Å (hydrazide for **L2**), O–H = 0.82 Å (hydroxyl for **L4**), and O–H = 0.84 Å (hydrogens in water for **L3**) with hydrogen‘s temperature factors U_iso_(H) = 1.2 U_eq_(C,N,O). The *DIAMOND* program package was used for molecular structure drawing [48]. Cambridge Crystallographic Data Centre (CCDC) 1498952–1498955 contains the supplementary crystallographic data for this article. These data can be obtained free of charge via www.ccdc.cam.ac.uk/conts/retrieving.html, or from the Cambridge Crystallographic Data Centre, 12 Union Road, CambridgeCB2 1EZ, UK; fax: +44-1223-336-033; or e-mail: deposit@ccdc.cam.ac.uk.

### 3.5. Urease Inhibition Assay

Both ligands and their copper(II) complexes were tested for their urease inhibition. The measurement of urease inhibitory activities was carried out using the modified method reported by Tanaka et al. [49]. Generally, the assay mixture containing 75 µL of jack bean urease and 75 µL of tested compounds (dissolved in DMSO) with various concentrations was pre-incubated for 15 min on a 96-well assay plate. Afterwards, 75 µL of phosphate buffer at pH 6.8 containing phenol red (0.18 mM) and urea (400 mM) were added and the mixture was incubated at room temperature. The reaction time required for enough ammonium carbonate to form to raise the pH phosphate buffer from 6.8 to 7.7 was determined by Biotek Synergy HT micro-plate reader (560 nm) with end-point indicated by the colour change of the phenol-red indicator. Acetohydroxamic acid was used as the standard reference substance.

### 3.6. Determination of Antimicrobial Activity

For testing of antimicrobial activity, bacteria *E. coli* (CNCTC 327/73), *S. aureus* (CNCTC Mau 82/78), and yeast *C. albicans* (CNCTC 59/91) were used. Bacteria were grown aerobically in nutrient broth (NB, Imuna, Šarišské Michaľany, Slovakia) and yeast in Sabouraud dextrose broth (SDB, Difco, Bordeaux, France) for 18 h at 37 °C or 48 h at 24 °C, respectively. Cultures were then maintained at 4 °C on appropriate solid medium: Endo agar (EA, Oxoid, Basingstoke, UK) for *E. coli*, blood agar (BA, Biomark, Pune, India) for *S. aureus*, and Sabouraud dextrose agar (SDA, Difco) for *C. albicans*. Working cultures were prepared by incubation of a single colony of each microorganism in NB (bacteria) or SDB (yeast) for 18 h at 37 °C or 48 h at 24 °C, respectively. A microbial suspension was prepared in saline solution (0.85% NaCl) according to the McFarland standard No. 0.5 using Lambda 35 UV/VIS Spectrophotometer (PerkinElmer, Waltham, MA, USA), to obtain a concentration of ca. 1.5 × 10^8^ cfu/cm^3^. After dilution in an appropriate liquid medium (NB for bacteria and SDB for yeast), a working concentration 1.5 × 10^7^ cfu/cm^3^ was prepared.

The antimicrobial activity assay was performed according to Bilková et al. [50] with some modifications. Stock solutions (200 mM) of test compounds and the standards (isoniazid and ciprofloxacin) were prepared in DMSO immediately before use. Working test compounds and the standards were prepared by serial dilution of stock solutions in sterile doubly concentrated NB or SDB to a final volume 100 mL within the 96-well microplates. Freshly prepared inoculum (5 mL) of the tested microorganism was added to each appropriate well (bacteria into the plates with NB, *C. albicans* into the plates with SDB). The final concentration of each organism in each well was ca. 7.5 × 10^5^ cfu/cm^3^. The concentration of tested compounds ranged from 100 mM to 0.2 μM, except for compounds **L1** and **L2**, which were tested in a range from 75 mM to 0.2 μM due to their lower available amount. Each concentration was tested in triplicate. For each assayed sample and microorganism, the following controls were applied: blank, uninoculated media without test compound to account for changes in the media during the experiment; negative control, uninoculated media containing only the test compound; positive control 1, inoculated media without compound, the positive control 2, inoculated media with serial dilution of DMSO without test compound, thereby assessing any activity of the solvent. The 96-well plates were incubated aerobically for 24 h at 37 °C or 24 °C, depending on whether bacteria or yeast were grown, respectively. Afterwards, 5 mL of each well was inoculated on the corresponding agar plate (BA for *S. aureus*, EA for *E. coli*, and SDA for *C. albicans*). Bacteria and yeast were grown aerobically for 24 h at 37 °C or 24 °C, respectively. The MIC was expressed as the lowest concentration of the compound inhibiting growth of the microorganism on the agar plates for all parallel samples in comparison with that of the positive control after 24 h.

## 4. Conclusions

Six Schiff base ligands and four copper(II) complexes from these ligands were prepared and characterized. The X-ray crystallographic analysis of four Schiff bases was performed and their spatial structure determined in solid state. Interestingly, the ligands are not planar, showing varying degrees of distortion of the conjugated system. The Schiff bases **L1** and **L4** exhibited interesting in vitro activity against *C. albicans*. All four copper(II) complexes showed excellent inhibition of Jack bean urease.

## Figures and Tables

**Table 1:** Selected bond distances (Å) and angles (°) for hydrazide group of ligands (**L1**–**L4**).

Ligand	L1	L2	L3	L4
Bond distances				
C1–N2	1.3509(17)	1.353(2)	1.347(2)	1.3500(17)
N2–N3	1.3800(15)	1.3679(18)	1.389(2)	1.3786(14)
N3–C7	1.2690(18)	1.270(2)	1.273(3)	1.2764(17)
Torsion angles				
C1–N2–N3–C7	169.57(13)	−173.01(16)	−171.94(19)	−176.96(11)

**Table 2:** Hydrogen-bond (interaction) geometry of ligands (**L1**–**L4**).

Ligand	D–H...A	d(D–H)	d(H...A)	d(D...A)	<(DHA)
**L1**	C7–H7A...O1 ^i^	0.93	2.45	3.2591(16)	145.4
	N2–H2A... O1 ^i^	0.86	2.15	3.9797(14)	161.0
**L2**	C7–H7...F2	0.93	2.46	2.980(2)	115.4
	C7–H7...F5	0.93	2.10	2.757(13)	126.6
	N2–H2N...N1 ^i^	0.89(2)	2.17(2)	3.0482(18)	172.5(19)
**L3**	O2–H2A...O1	0.8396(19)	2.274(16)	2.954(2)	138(2)
	O2–H2A...N3	0.8396(19)	2.565(14)	3.271(2)	142(2)
	C7–H7...O2 ^i^	0.93	2.64	3.338(2)	132.3
	N2–H2...O2 ^i^	0.86	2.02	2.834(2)	157.8
	O2–H2B...N1 ^ii^	0.835(18)	2.06(2)	2.861(2)	161(2)
	C4–H4...F1 ^iii^	0.93	2.55	3.349(3)	144.6
**L4**	O2–H2O...N3	0.82	1.92	2.6316(15)	145.3
	C4–H4...F1 ^i^	0.93	2.64	3.281(2)	126.9
	C12–H12...O1 ^ii^	0.93	2.51	3.2566(18)	138.1
	N2–H2N...N1 ^iii^	0.86	2.19	2.8861(15)	138.1

For **L1**: (i) x, y − 1, z; for **L2**: (i) x + 1, y, z; for **L3**: (i) x − 1, y, z; (ii) −x + 1, y + 1/2, −z + 1/2; (iii) –x + 3/2, −y + 1, z − 1/2; for **L4**: (i) x + 1,y + 1, z; (ii) –x + 1, y − 1/2, −z + 1/2; (iii) –x + 2, y − 1/2, −z + 3/2.

**Table 3:** Antimicrobial bioassay (minimum inhibitory concentration (MIC) values) for ligands and Cu(II) complexes.

	Compound	*S. aureus* Mau 82/78	*E. coli* 327/73	*C. Albicans* 59/91
MIC [mM]	**L1**	37.5	9.37	0.037
	**L2**	37.5	18.76	9.37
	**L3**	n.i.	25.0	25.0
	**L4**	n.i.	1.55	0.048
	**L5**	50.0	25.0	12.5
	**L6**	50.0	12.5	12.5
	**Cu-L3**	6.25	6.25	6.25
	**Cu-L1**	6.25	12.5	6.25
	**Cu-L2**	12.5	12.5	12.5
	**Cu-L6**	0.76	6.25	6.25
	Isoniazid	n.i.	n.i.	50.0
	Ciprofloxacin	6.8 × 10^−4^	<3 × 10^−4^	n.a.

n.a. = not applicable, n.i. = no inhibition observed at the highest used concentration (100 mM).

**Table 4:** Inhibition of jack bean urease by ligands and Cu(II) complexes.

Compounds	IC_50_ ± SD (μM)
ligands	>500
**Cu-L3**	1.17 ± 0.02
**Cu-L1**	0.49 ± 0.01
**Cu-L2**	1.25 ± 0.03
**Cu-L6**	1.01 ± 0.02
Acetohydroxamic acid	185 ± 6.2

**Table 5:** Crystal data and structure refinement for Schiff base ligands (**L1**–**L4**).

	L1	L2	L3	L4
Empirical formula	C_14_H_10_F_3_N_3_O	C_14_H_10_F_3_N_3_O	C_13_H_12_FN_3_O_2_	C_13_H_10_FN_3_O_2_
Temperature (K)	293(2) K	293(2) K	293(2) K	293(2) K
Wavelength (Å)	0.71073	1.54184	0.71073	0.71073
Crystal system	Monoclinic	Monoclinic	Orthorhombic	Monoclinic
Space group	P2_1_/n	P2_1_/c	P2_1_2_1_2_1_	P2_1_/c
Unit cell dimensions	a = 11.7577(7) Å	a = 8.1335(2) Å	a = 6.4785(4) Å	a = 8.9048(3) Å
	b = 5.1630(2) Å	b = 7.2718(2) Å	b = 7.0244(10) Å	b = 10.1067(3) Å
	c = 21.6108(13) Å	c = 22.2575(6) Å	c = 27.373(4) Å	c = 13.6087(5) Å
	a = 90°	a = 90°	a = 90°	a = 90°
	b = 98.143(5)°	b = 98.686(2)°	b = 90°	b = 103.420(4)°
	g = 90°	g = 90°	g = 90°	g = 90°
Formula weight	293.25	293.25	261.26	259.24
Volume (Å^3^)	1298.66(12)	1301.33(6)	1245.7(3)	1191.32(7)
Z/Calculated density (mg/m^3^)	4/1.500	4/1.497	4/1.393	4/1.445
Absorption coefficient (mm^−1^)	0.127	1.101	0.107	0.111
F (000)	600	600	544	536
Crystal size (mm)	0.97 × 0.78 × 0.21	0.30 × 0.15 × 0.05	0.37 × 0.11 × 0.08	0.62 × 0.23 × 0.12
Theta range for data collection	3.132° to 26.361°	4.018° to 75.559°	2.977° to 30.497°	3.097° to 26.368°
Index ranges	−14 ≤ h ≤ 14	−10 ≤ h ≤ 10	−9 ≤ h ≤ 9	−11 ≤ h ≤ 11
	−6 ≤ k ≤ 6	−9 ≤ k ≤ 9	−10 ≤ k ≤ 10	−12 ≤ k ≤ 12
	−27 ≤ l ≤ 27	−27 ≤ l ≤ 27	−39 ≤ l ≤ 39	−16 ≤ l ≤ 16
Reflections collected	19468	19144	27502	18517
Independent reflections	2654 [R(int) = 0.0218]	2675 [R(int) = 0.0418]	3797 [R(int) = 0.0352]	2432 [R(int) = 0.0292]
Completeness to 2*Θ* = 25.000°	99.7%	100.0%	99.9%	99.8%
Refinement method	Full-matrix least-squares on F^2^	Full-matrix least-squares on F^2^	Full-matrix least-squares on F^2^	Full-matrix least-squares on F^2^
Data/restraints/parameters	2654/268/218	2675/268/221	3797/3/178	2432/0/174
Goodness-of-fit on F^2^	1.089	1.034	1.015	1.066
Final R indices (I > 2σ(I))	R1 = 0.0405	R1 = 0.0410	R1 = 0.0428	R1 = 0.0382
	wR2 = 0.1063	wR2 = 0.1133	wR2 = 0.0946	wR2 = 0.1035
R indices (all data)	R1 = 0.0474	R1 = 0.0530	R1 = 0.0633	R1 = 0.0461
	wR2 = 0.1120	wR2 = 0.1227	wR2 = 0.1040	wR2 = 0.1097
Extinction coefficient	0.023(4)	n/a	n/a	0.012(2)
Largest difference peak and hole (e·Å^−3^)	0.177 and −0.189	0.181 and −0.254	0.202 and −0.145	0.245 and −0.239
